# Selective Activation of Resting-State Networks following Focal Stimulation in a Connectome-Based Network Model of the Human Brain

**DOI:** 10.1523/ENEURO.0068-16.2016

**Published:** 2016-10-06

**Authors:** Andreas Spiegler, Enrique C. A. Hansen, Christophe Bernard, Anthony R. McIntosh, Viktor K. Jirsa

**Affiliations:** 1 Institut de la Santé et de la Recherche Médical, Institut de Neurosciences des Systèmes UMR_S 1106, Aix Marseille Univ., INSERM, INS, Inst. Neurosci. Syst., 13005 Marseille, France; 2 Rotman Research Institute of Baycrest Center, University of Toronto, Toronto, Ontario M6A 2E1, Canada

**Keywords:** connectivity, connectome, criticality, network modeling, resting state, stimulation

## Abstract

When the brain is stimulated, for example, by sensory inputs or goal-oriented tasks, the brain initially responds with activities in specific areas. The subsequent pattern formation of functional networks is constrained by the structural connectivity (SC) of the brain. The extent to which information is processed over short- or long-range SC is unclear. Whole-brain models based on long-range axonal connections, for example, can partly describe measured functional connectivity dynamics at rest. Here, we study the effect of SC on the network response to stimulation. We use a human whole-brain network model comprising long- and short-range connections. We systematically activate each cortical or thalamic area, and investigate the network response as a function of its short- and long-range SC. We show that when the brain is operating at the edge of criticality, stimulation causes a cascade of network recruitments, collapsing onto a smaller space that is partly constrained by SC. We found both short- and long-range SC essential to reproduce experimental results. In particular, the stimulation of specific areas results in the activation of one or more resting-state networks. We suggest that the stimulus-induced brain activity, which may indicate information and cognitive processing, follows specific routes imposed by structural networks explaining the emergence of functional networks. We provide a lookup table linking stimulation targets and functional network activations, which potentially can be useful in diagnostics and treatments with brain stimulation.

## Significance Statement

Systematic exploration via stimulation of all cortical and subcortical brain areas can only be performed *in silico*. We have performed a detailed parametric exploration of dynamically responsive networks of a large-scale brain network model of stimulation and developed a stimulation map indicating which brain areas need to be stimulated to place the brain in a particular state at rest. Brain stimulation is one of the upcoming novel tools in the treatment of neurological disorders. The stimulation map will be critical in guiding these studies and will allow for the development of theory-guided stimulation protocols.

## Introduction

Sensory stimulation is important to understand perception and information processing in the brain. To study cognitive functions, direct stimulation techniques, such as transcranial magnetic stimulation (TMS) and transcranial electrical stimulation, are increasingly used. Moreover, direct brain stimulation is promising for treating psychiatric and neurological disorders (Parkin et al., 2015). The effects of direct stimulation are short range (i.e., local in a brain region) and long range (i.e., on a large-scale network). Both are important to understand the final outcome of the stimulation (Fox et al., 2014). There is, however, scant knowledge regarding the way of stimulating the brain to cause a predictable and beneficial effect. A conceptual framework is missing. Furthermore, the extent to which information is processed over short or long ranges is unclear.

Brain structures bear dynamics that give rise to diverse function and dysfunction (e.g., Park and Friston, 2013). Because structural connectivity (SC) constrains functional networks (Deco et al., 2015), we predict that stimulating a given area will give rise to a process of activity, ultimately resolving in spatial patterns resembling functionally related networks. For example, direct stimulation of a primary sensory structure (e.g., the nucleus geniculatus lateralis thalami for the visual pathway) should cause responsive networks similar to those activated by a (visual) sensory input. The stimulation site of a responsive network can be part of (1) functional networks in which information is processed, (2) ascending paths of sensory inputs, and (3) structures modulating the information processing. Testing this hypothesis experimentally is delicate, as it requires knowing where and how to stimulate. The effect of stimulation of various cortical and subcortical brain areas can be systematically explored *in silico*.

Here, we use The Virtual Brain (TVB) platform, which allows studying dynamics in whole-brain models (Sanz-Leon et al., 2013, 2015), to systematically stimulate every area in the network comprising long- and short-range SC (i.e., between brain areas and within an area), detect the responsive networks, and then contrast these to experimentally known networks, especially the resting-state (RS) networks (Damoiseaux et al., 2006). RS networks describe, in the absence of external inputs or goal-oriented tasks, the consistent spatial patterns in the fluctuations of the BOLD signal (functional MRI). Furthermore, these patterns have been correlated to functionally related brain regions (i.e., active during task conditions) and have been called, for example, visual, memory, and attention RS networks. However, the link between the RS networks and the functional networks occurring due to external stimuli or during goal-oriented tasks is not clear. The RS networks, moreover, correlate with the SC of white matter tracts (Greicius et al., 2009; van den Heuvel et al., 2009; Hermundstad et al., 2013), thus appear as simple reflections of the large-scale network topology.

Local and global computation in the brain strongly depends upon short-range and long-range structural connections (Deco et al., 2015). We are taking into account both types of SC in TVB. Previous large-scale network model studies mostly considered long-range SC (i.e., white matter tracts). We go beyond this and incorporate short-range SC to understand how activity propagates and dissipates in the brain (Jirsa and Kelso, 2000; Jirsa, 2004; Qubbaj and Jirsa, 2007, 2009).

Large-scale brain networks have specific constraints due to the spatiotemporal scale of operation. First, the time delays due to signal transmission via long white matter tracts between connecting nodes in brain network dynamics play a crucial role, for instance, in the generation of ongoing activity (Ghosh et al., 2008). Second, the connection strength, when scaled appropriately, places the brain close to criticality, where the capacity of processing information is maximized and the functional connectivity best fits to empirical RS data (Ghosh et al., 2008; Deco and Jirsa, 2012; Deco et al., 2014). Finally, random processes serve to provide the brain model with kinetic energy to form and alter functional networks (Ghosh et al., 2008; Deco et al., 2014; Hansen et al., 2015).

Using an unbiased and deterministic approach, here we demonstrate the large-scale brain network response to stimulation with functionally relevant activity patterns, which resemble the experimentally known RS networks. In particular, we show that stimulation at spatially distant sites can give rise to similar nonstationary trajectories, whereas stimulation at spatially close sites can result in distinctly different dynamics.

## Materials and Methods

**Figure 1.**
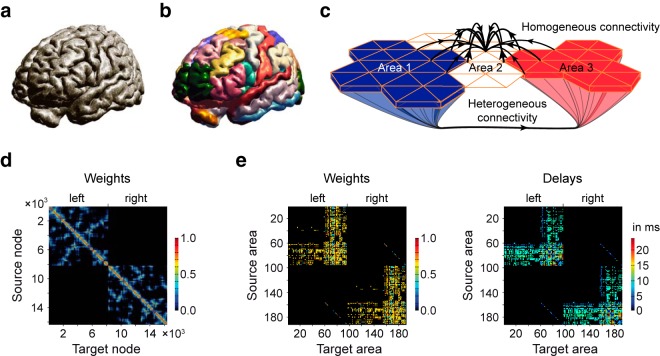
Structure of the large-scale brain model. ***a***, The large-scale brain model is composed of the geometry of the brain of 116 subcortical areas and the two cerebral hemispheres. ***b***, There are 37 cortical areas, each containing between 29 and 683 nodes (dots in ***a***), for a total of 8,192 nodes per hemisphere. ***c***, Homogeneous and heterogeneous SC. Heterogeneous SC corresponds to white matter tracts connecting brain areas over long distances. Homogeneous SC corresponds to gray matter fibers, with short-range connections within a given area, but also enabling some communication over short distances between neighboring areas. Although Area 2 is not connected to Areas 1 and 3 via the white matter, it is weakly linked to both areas via a set of short-range SC. ***d***, Homogeneous SC matrix for the 16,384 nodes. The synaptic weights are color coded. The diagonal describes in warm colors the strong SC of adjacent nodes. SC decreases with distance, which is shown in cold colors. SC of nearby nodes are scattered (e.g., blue dots) in ***d*** because each cerebral hemisphere is described by a surface, which makes it impossible to cluster nodes locally along both axes. Note the absence on interhemispheric short-range SC. ***e***, Heterogeneous SC for the 190 (74 cortical plus 116 subcortical) areas for weights (left) and time delays (right). Within one hemisphere, the 58 subcortical areas mostly project to the 37 cortical areas. Some connections between subcortical areas can also be seen. The 37 cortical areas project heavily to both cortical and subcortical areas. Some interhemispheric connections can also been seen. Note also the presence of large time delays.

Using The Virtual Brain platform (Sanz-Leon et al., 2013, 2015), we triangulate the surface of the cortex with a mesh of 8,192 nodes for each hemisphere (Fig. 1*a*), distributed across 74 cortical areas (Fig. 1*b*), each containing between 29 and 683 nodes (Table 1), following a known functional parcellation atlas (Kötter and Wanke, 2005). The model also includes 116 nonparcellated subcortical areas. To connect nodes with each other, we distinguish homogeneous from heterogeneous SC (Fig. 1*c–e*). The homogenous SC (of short-range connections) links nodes within an area, and between areas if they are spatially close to one another with a connection probability decreasing with distance (Braitenberg and Schüz, 1991; Fig. 1*c*,*d*). The heterogeneous SC (of long-range white matter tracts) links all the nodes of an area with the nodes of another area (Fig. 1*c*,*e*), based on known anatomical data (Kötter and Wanke, 2005). Neighboring areas are able to exchange information via the homogeneous SC within the cortex and via the white matter tract, that is, the heterogeneous SC (Fig. 1*c*, Area 2 with Areas 1 and 3).

**Table 1:** Abbreviations of brain areas

A1	Primary auditory cortex (57,74)	Cld	Capsule of the nucleus lateralis dorsalis
A2	Secondary auditory cortex (33,64)	CnMd	Nucleus centrum medianum thalami
Amyg	Amygdala (151,135)	Cs	Nucleus centralis superior thalami
CCa	Gyrus cinguli anterior (54,49)	Csl	Nucleus centralis superior lateralis thalami
CCp	Gyrus cinguli posterior (167,179)	GL	Nucleus geniculatus lateralis thalami
CCr	Gyrus cinguli retrosplenialis (68,67)	GM	Nucleus geniculatus medialis thalami
CCs	Gyrus cinguli subgenualis (29,42)	GMpc	Nucleus geniculatus medialis thalami, pars parvocellularis
FEF	Frontal eye field (104,161)	IL	Intralaminar nuclei of the thalamus
G	Gustatory cortex (52,42)	LD	Laterodorsal nucleus (thalamus)
HC	Hippocampal cortex (75,54)	Li	Nucleus limitans thalami
Ia	Anterior insula (48,71)	LP	Nucleus lateralis posterior thalami
Ip	Posterior insula (82,111)	MD	Nucleus medialis dorsalis thalami
M1	Primary motor area (463,460)	MDdc	Nucleus medialis dorsalis thalami, pars densocellularis
PCi	Inferior parietal cortex (454,371)	MDmc	Nucleus medialis dorsalis thalami, pars magnocellularis
PCip	Cortex of the intraparietal sulcus (355,486)	MDmf	Nucleus medialis dorsalis thalami, pars multiformis
PCm	Medial parietal cortex (196,241)	MDpc	Nucleus medialis dorsalis thalami, pars parvocellularis
PCs	Superior parietal cortex (199,177)	ML	Midline nuclei of the thalamus
PFCcl	Centrolateral prefrontal cortex (328,227)	Pa	Nucleus paraventricularis thalami
PFCdl	Dorsolateral prefrontal cortex (248,216)	Pac	Nucleus paraventricularis caudalis thalami
PFCdm	Dorsomedial prefrontal cortex (211,270)	Pcn	Nucleus paracentralis thalami
PFCm	Medial prefrontal cortex (61,68)	Pf	Nucleus parafascicularis thalami
PFCorb	Orbital prefrontal cortex (310,265)	PT	Nucleus parataenialis thalami
PFCpol	Pole of prefrontal cortex (279,279)	Pul	Nucleus pulvinaris thalami
PFCvl	Ventrolateral prefrontal cortex (380,479)	Pul.i	Nucleus pulvinaris inferior thalami
PHC	Parahippocampal cortex (267,212)	lPul.l	Nucleus pulvinaris lateralis thalami
PMCdl	Dorsolateral premotor cortex (108,138)	Pul.m	Nucleus pulvinaris medialis thalami
PMCm	Medial premotor cortex (149,68)	Pul.o	Nucleus pulvinaris oralis thalami
PMCvl	Ventrolateral premotor cortex (126,138)	R	Nucleus reticularis thalami
S1	Primary somatosensory cortex (487,420)	Re	Nucleus reuniens thalami
S2	Secondary somatosensory cortex (107,116)	SG	Nucleus suprageniculatus thalami
TCc	Central temporal cortex (436,422)	Teg.a	Nucleus tegmentalis anterior
TCi	Inferior temporal cortex (390,306)	VA	ventral anterior nucleus (thalamus)
TCpol	Pole of temporal cortex (91,101)	VAmc	Nucleus ventralis anterior thalami, pars magnocellularis
TCs	Superior temporal cortex (306,352)	VApc	Nucleus ventralis anterior thalami, pars parvocellularis
TCv	Ventral temporal cortex (260,317)	VL	ventral lateral nucleus (thalamus)
V1	Visual area 1 (147,180)	VLc	Nucleus ventralis lateralis thalami, pars caudalis
V2	Secondary visual cortex (683,663)	VLm	Nucleus ventralis lateralis thalami, pars medialis
		VLo	Nucleus ventralis lateralis thalami, pars oralis
AD	Nucleus anterior dorsalis thalami	VLps	Nucleus ventralis lateralis thalami, pars postrema
AM	Nucleus anterior medialis thalami	VP	Nucleus ventralis posterior
AN	Anterior nuclei of the thalamus	VPI	Nucleus ventralis posterior inferior thalami
AV	Nucleus anterior ventralis thalami	VPL	Aentral posterior lateral nucleus (thalamus)
Caud	Nucleus caudatus	VPLc	Nucleus ventralis posterior lateralis thalami, pars caudalis
Cdc	Nucleus centralis densocellularis thalami	VPLo	Nucleus ventralis posterior lateralis thalami, pars oralis
Cif	Nucleus centralis inferior thalami	VPM	Nucleus ventralis posterior medialis thalami
Cim	Nucleus centralis intermedialis thalami	VPMpc	Nucleus ventralis posterior medialis, pars parvocellularis
Cl	Nucleus centralis lateralis thalami	X	Area X (thalamus)
Clau	Claustrum	Clc	Nucleus centralis latocellularis thalami

Number of nodes per cortical areas in brackets (left, right).

Each vertex point is a network node holding a neural mass model connected to other nodes via the homogeneous SC and heterogeneous SC. When an area is stimulated, all the nodes of this area are simultaneously activated, and then the stimulation-induced activity in each node decays differently according to the activity in the surrounding area via short-range connections (i.e., homogeneous SC) and remote nodes via long-range connections (i.e., heterogeneous SC). The ability to drive the network does not depend on the number of nodes within an area, because the heterogeneous SC transfers the mean of the activity in all of the nodes within an area to all the nodes in another area.

We consider this ratio of homogeneous SC to heterogeneous SC as a degree of freedom and performed a parametric study (for systematic studies with two-point connection, see Jirsa and Kelso, 2000; Qubbaj and Jirsa, 2007, 2009). The ratio has been estimated. For instance, Braitenberg and Schüz (1998) assessed that pyramidal cells have synapses in equal shares from long-range and local axons. However, the ratio of homogeneous SC to heterogeneous SC mainly depends on the resolution of the used geometrical model of the cortex, and with that the representation of the SC, and the network node description (e.g., canonical model, neural mass model), which is able to incorporate local connectivity (for more detail, see Spiegler and Jirsa, 2013). At the extremes, (1) 0% of heterogeneous SC (thus, 100% of homogeneous SC gives two unconnected cerebral hemispheres with locally but homogeneously connected nodes) only allows activity to propagate locally from a cortical stimulation site, and (2) 100% of heterogeneous SC (thus 0% of homogenous SC gives 190 purely heterogeneously connected brain areas with locally unconnected nodes) only allows activity to travel long distances with time delays via white matter fiber tracts.

Furthermore, since the spatial range of homogeneous SC is not known (Spiegler and Jirsa, 2013), we also consider it as a parameter varying between 10 and 41 mm. We then systematically stimulate each of the 190 areas with a large range of parameter values (for the ratio and the spatial range), resulting in a total of all 37,620 simulation trials.

**Figure 2.**
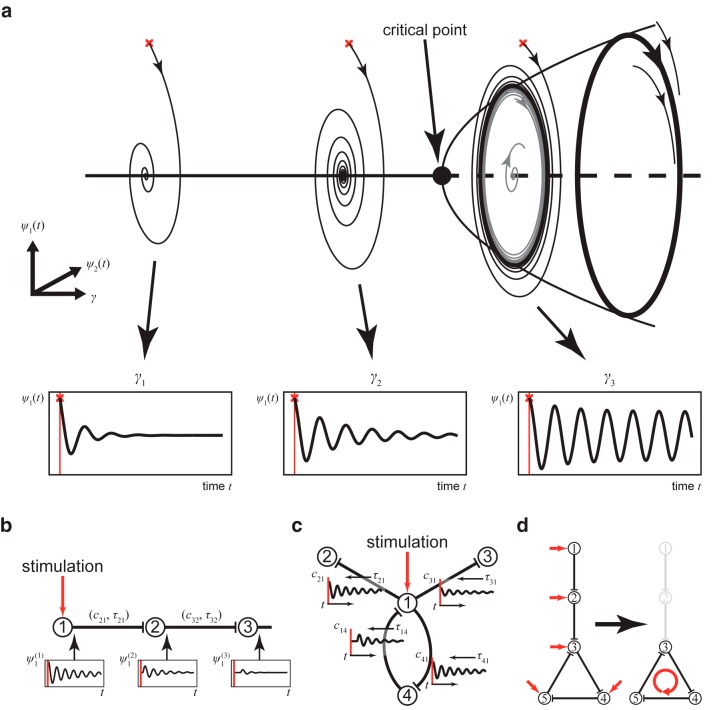
The large-scale brain model works near criticality. ***a***, Each node in the model is parameterized by *γ* to operate intrinsically at the same distance from the critical point if unconnected. A node shows zero activity or oscillation (∼42 Hz) in response to stimulation (red crosses). The activity at each node is described by two time-dependent variables, *ψ*_1_(*t*) and *ψ*_2_(*t*). The closer a node operates to the critical point, the larger and the longer lasting is the oscillation (compare *γ*_1_ and, *γ*_2_). When the critical point is reached, the node intrinsically performs a rhythm of constant magnitude. The model, however, is set so that the critical point is never exceeded. ***b***, Principles of activity spreading after stimulation. The damped oscillation generated in the stimulated node (1) is sent via its efferent connections to its target node (2), triggering there, in turn, a damped oscillation with weaker amplitude and faster decay, which then propagates to the next node. Activity *ψ*_1_^(^*^j^* ^)^ (*t*) of node (*j*) is scaled by *c_ij_* and transmitted to node (*i*) via homogeneous and heterogeneous connections (SCs), delayed by *τ_ij_* in the latter case. In such a chain, activity would decay fast. ***c***, In the large-scale brain model, multiple activity re-entry points can be found. At any time point, the dynamics of a node is influenced by all incoming activity. The response of the node to stimulation (1) is relayed to linked nodes (2–4), which may be fed back to 1 via 4 and may allow the induced activity to dissipate on a much longer time scale. The network response thus depends upon the SC and allows the network to operate near criticality. ***d***, Activation of dynamically responsive networks. Activity after stimulating a node (1 or 2) in a series connection decays fast (as in ***b***). However, activity may circulate and thus decays slower in a feedback network (4–5). Such remaining activity after the initial stimulation decay reveals the so-called dynamically responsive networks.

Brain dynamics at rest have been found to operate near criticality (Ghosh et al., 2008; Deco et al., 2011, 2013). Near criticality is defined as a system that is on the brink of a qualitative change in its behavior (Shew and Plenz, 2013). The proximity to criticality predicts that the response of the brain to stimulation will primarily arise from structures and networks that are closest to instability. Activities in those networks require the most time to settle into equilibria after stimulation, and are associated with large-scale dependencies and scale invariance (Haken, 1978). This would be consistent with the center manifold theorem, which states that a high-dimensional system in a subcritical state will converge on a lower-dimensional manifold (few networks) when the system is stimulated. Consequently, we equally set each node in the brain network model to operate close to its critical point, where the network shows no activity without stimulation. We use the stable regimen of each network node (i.e., stable focus) to stimulate a given area in the direction of its instability point (i.e., supercritical Andronov–Hopf bifurcation) and to induce characteristic energy dissipation through the brain network. The dissipation of energy will be constrained by the homogeneous SC and heterogeneous SC, the associated signal transmission delays, and the local dynamics at the network nodes. In the network model, the operating point of every node, when disconnected from the network, is at the same distance from its critical point, that is, the supercritical Andronov–Hopf bifurcation (Fig. 2*a*). If the critical point is reached, the node enters into a constant oscillatory mode. In the network, the SC (including time delays) determines the alteration of the working distance to the critical point at each node in time by weighting and delaying the incoming activity from other nodes in the network. Hence, network metrics of the SC such as the in-strength, that is, the sum of weights of incoming ties to a node may indicate the distance of the operating point of a node to its critical point, and, thus, the criticality (Kunze et al., 2016). The network model, however, is set so that criticality is never reached by normalizing the SC to unity maximum in-strength so that activity cannot be amplified through the SC. As a result, when a node is stimulated, the node operates closer to the critical point, and the response is in the form of a damped oscillation (Fig. 2*a*). The closer a node operates to the critical point, the stronger the responses of the node with high amplitude and long decay time (Fig. 2*a*). The nodes are working near criticality (i.e., they get close to a change in behavior, which would here be a switch to a constant oscillatory mode, but never reaching it). Thus, the response to the stimulation is transient, lasting a few milliseconds. The damped oscillation generated in one stimulated node is then sent via its efferent connections to its target nodes, triggering there, in turn, a damped oscillation (Fig. 2*b*). If the network were based mainly on nodes connected in series, activity would decay very fast after the stimulation (Fig. 2*b*). However, since the outgoing activity of a node can influence the nodes projecting back to it, recurrent systems appear (Fig. 2*c*,*d*) that allow activity to dissipate on a much longer time scale. The evoked activity, after the initial decay, thus persists in the so-called responsive networks (Fig. 2*c*,*d*), which may reflect feedback loops and re-entry points in the SC. A dynamically responsive network acts on changes, for instance, those due to sensory stimuli and random fluctuations in the network (flexibility), and outlasts the stimulation (criticality).

**Figure 3.**
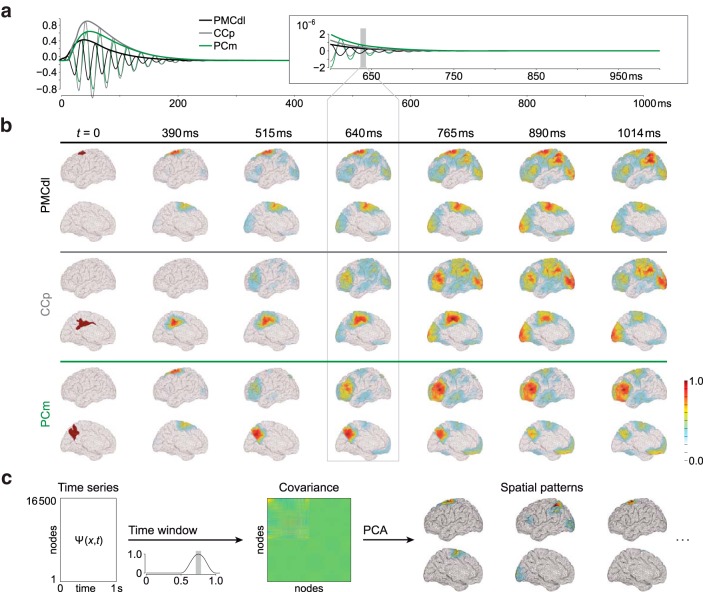
Dissipation after stimulation. ***a***, Response of area PFCcl to the activation of three different regions PMCdl, CCp, and PCm (abbreviations are given in Table 1). Note that the amplitude, decay, and phase of the response depend upon the stimulated area. The main determinants of the response pattern are the connections, the synaptic weights, and the time delays. The envelope of the time series is computed (black, gray, and green lines for the three stimulation sites). ***b***, Spatiotemporal activation following stimulation of three different regions. At a given time point, we extract the amplitude of the envelope for the 16,500 nodes (the 16,384 cortical nodes and the 116 subcortical ones), which we normalize to 1. The color scale thus indicates the contribution of a given region to the overall activity. The dissipation of activity after stimulating two distant brain areas, PMCdl and CCp (located far from one another: PMCdl in the lateral surface, CCp in the medial surface) leads to similar topographical patterns (for *t* > 640 ms). In contrast, a distinct pattern appears when stimulating PCm, which is adjacent to CCp. ***c***, Extraction of the main activated propagation subnetworks. We use the stimulation of PMCdl as an example. We calculate the covariance among the 16,500 time series (the 16,384 cortical nodes and the 116 subcortical ones) for a time window centered at 750 ms and then perform a PCA to extract the subnetworks capturing >99% of the activity. Three different networks are thus dynamically responsive when PMCdl is stimulated.

The described network properties are illustrated in Figure 3*a*. The stimulation of three different areas gives rise to three different responses in a given target area. The differences stem from the proximity to criticality, which depends upon the SC (in particular, the extent of recurrent networks), comprising the synaptic weights and the time delays (Fig. 1). This behavior is predicted by the center manifold theorem, which is the mathematical basis for criticality (Haken, 1978).

### Large-scale brain model

Dynamics of a vector field Ψ (*x*, *t*) at time *t* ∈ **R**^1^ and position *x* ∈ **R**^3^ in space Ω are described by a delay-integro-differential equation, as follows:

(1) $$\partial_{t}\Psi \,\;(x,t)=E\,\;(\Psi \,\;(x,t))-a_{I}\;I(x,t)+(1-\alpha )\;a_{L}\underset{L}{\int }dX\prime \;\;\;\Psi \,\;\,(x-X\prime ,t)\;g(X\prime )+\alpha \,\;\;a_{s}\,\;\underset{\Omega }{\int }dX\prime \;\;\;\Psi \,\;(x-X\prime ,\;t-\|x-X\prime \|\;/v)\times H(x)\;C(\|x-X\prime \|\;/v)\;\;K^{T}\,\;(X\prime ),$$

where ∂*_t_* is the derivative with respect to time *t*. The input *I*(*x*, *t*) allows the stimulation dynamics to intervene on a node. The operator *E* (Ψ (*x*, *t*)) locally links variables of the vector field. The scalar *α* balances the effect of the homogeneous SC and the heterogeneous SC (first and second integrals) on the vector field. The vectors *a_I_*, *a_L_*, and *a_S_* of factors relate to the input *I*, and both types of SC to the vector field Ψ (*x*, *t*). The kernel *g*(*x*) describes the homogeneous SC. The field is time delayed due to a finite transmission speed, *v*, via the heterogeneous SC given by matrix *C*(*x*). The vectors *H*(*x*) and *K*(*x*) establish the links between the heterogeneous SC and the targets and sources. Note that the transmission speed enters the second integral concerning heterogeneous SC. We assumed the transmission via the homogeneous SC (first integral) to be instantaneous, which reduces the computational expenses, in order to perform the parameter study. The spatial and temporal aspects of the model are described in more detail in the following two subsections.

### Geometry and SC

The spatial domain Ω = {*L*_1_ ∪ *L*_2_ ∪ *S*} separates both cerebral hemispheres *L* = {*L*_1_ ∪ *L*_2_}: left, *L*_1_ and right, *L*_2_, from subcortical areas *S*, that is, ∩Ω = ∅. A closed 2-sphere describes the geometry of each hemisphere (*L*_1_ and *L*_2_). The homogeneous SC follows a Gaussian distribution *g*(*x*) = exp(−*x*^2^/(2*σ*^2^)) that is invariant under translations on *L* (Spiegler and Jirsa, 2013). Each closed sphere, *L*_1_ and *L*_2_, is divided into *m* = 38 regions, that is, *L*_1_ = ∪_*r∈R*_1__ *A_r_* and *L*_2_ = ∪_*r∈R*_2__ *A_r_* with *R*_1_ = *R*(*m*), *R*_2_ = *R*_1_ + *n*: *R*(λ ∈ **N**) = {*r*|*r* ∈ **N**, *r*≤λ}, where *n* = 116 is the number of subcortical areas. The division of the spheres into regions follows a coarser Brodmann map (Kötter and Wanke, 2005) of areas, *A_r_* = *A* (*r* ∈ **N**) ∈ Ω: **N → R**^3^ onto space Ω for introducing heterogeneous SC (in default model in TVB; Sanz-Leon et al., 2013, 2015). The corpus callosum intersects the medial faces of both closed 2-spheres to interconnect both cerebral hemispheres from within, leaving two openings. All the nodes in the intersecting regions are placed far enough so that the nodes are topologically isolated by *g*(*x* − *X′*) → 0. Finally, one region is the intersection by the corpus callosum, and the remaining regions are the considered 37 cortical areas composing a cerebral hemisphere. Each of the *n* = 116 considered subcortical areas is lumped to a single point in space *S* = ∪_*r∈R*_3__ *A_r_* with *R*_3_ = *R*(*n*) + 2*m*. The heterogeneous connections *C* transmit mean activities of sources to target areas *H*(*x*) and *K*(*X′*) with a finite transmission speed, *v* = 6 ms^−1^ (Nunez, 1995, 1981). The square matrix *C* (‖ *x* − *X′* ‖ /*v*) contains (2*m* + *n*)^2^ weights and *c_ij_* (‖ *x* − *X′* ‖ /*v*): *i*, *j* = 1, . . . , 2*m* + *n* taken from the *CoCoMac* database (Stephan et al., 2001; Kötter, 2004; Kötter and Wanke, 2005), which was extrapolated to humans (Sanz-Leon et al., 2013, 2015). The row vectors *H*(*x*) and *K*(*X′*) contain 2*m* + *n* operations, *h_i_*(*x*) and *k_j_*(*X′*) on the targets and sources, respectively. The operations are *h_i_*(*x*) = *δ_x_*(*A_i_*) and *k_j_*(*X′*) = *δ_X′_*(*A_i_*) /|*A_j_*| with the Dirac measure *δ*_Ω_(*A*) on Ω and the cardinality |*A_r_*| of the set *A_r_*.

The description of the large-scale brain network model (Eq. 1) is fully compatible with previous TVB descriptions (Spiegler and Jirsa, 2013; Sanz-Leon et al., 2015). Note that the set notation is used here to describe brain areas and the division of homogeneously distributed and connected network nodes on both cerebral hemispheres into cerebral areas. This is novel here and has not been addressed in previous TVB publications.

### Temporal dynamics

The vector field describes a two-dimensional flow (Stefanescu and Jirsa, 2008) linking two variables Ψ (*x*, *t*) = (*ψ*_1_ *ψ*_2_)^T^ (*x*, *t*) in Equation 1, as follows:

(2) $$E\,\;(\Psi \,\;(x,t))=\eta (\begin{array}{c} \psi_{2}(x,t)-\gamma \,\;\psi_{1}(x,t)-\psi_{1}^{3}(x,t)\\ -\varepsilon \,\;\psi_{1}\,\;(x,t) \end{array}).$$

The parameterization *γ* = 1.21 and *ε* = 12.3083 sets an isolated brain area close to a critical point, that is, an Andronov–Hopf bifurcation (sketched in Fig. 2) with a natural frequency of ∼42 Hz using a characteristic rate of *η* = 76.74 s^−1^. This rhythm in the gamma band accounts for local activity, such as a coordinated interaction of excitation and inhibition (Buzsáki and Wang, 2012), which is not explicitly modeled here. The Dirac delta function is applied to a brain area, *I_r_* (*x*, *t*) = −5*η δ_x_*(*A_r_*) *δ*(*t*). The connectivities and the input act on the first variable *ψ*_1_(*x*, *t*) in Equation 1 by *a_L_* = *a_S_* = *a_I_*^T^ = (*η* 0). The connectivity-weighted input determines criticality by working against inherent energy dissipation (i.e., stable focus) toward the bifurcation. So that the bifurcation was not passed, both homogeneous and heterogeneous SC, *g*(*x*) and *C* (‖ x − *X′* ‖ /*v*), are normalized to unity maximum in-strength across time delays by (1) ∫ d*x g*(*x*) = 1 and (2) $\sup_{\lambda \in \Omega \,\;/\;v}\;\{\Sigma_{j}^{n}\,\;c_{ij}\,\;(\|\lambda \|)\}=1.$

### Simulation

To simulate the model on a computer, physical space and time are discretized. The folding of the human cortex presents a challenge for sampling. The cerebral surfaces, *L*_1_ and *L*_2_, are evenly filled with 8192 nodes. Subcortical structures in *S* remain unaffected by the discretization. The geometry of the brain is captured in physical space, Ω by a net of 16,500 nodes (i.e., 16,384 cortical and 116 subcortical nodes). The spatial integrals in Equation 1 are rewritten as matrix operations, where the heterogeneous SC remains the same and the homogeneous SC is spatially sampled on the cerebral surfaces (Spiegler and Jirsa, 2013). The system of difference equations is then solved using Heun’s method with a time step of 40 μs for 1 second/realization of one of the following factors: each of the 190 stimulation sites, SC balance, α = {0.0, 0.2, 0.4, 0.6, 0.8, 1.0}, and homogeneous spreading, σ/mm ∈ **N**: 10 ≤ σ/mm ≤ 41. The implementation is verified by the algebraic solution of an isolated node (i.e., no connections), and by the field properties (e.g., compact solutions spreading radially around a stimulation site) of the homogeneously linked cerebral nodes.

The lower bound of the spatial range of σ = 10 mm results from the geometrical model used for the cortex. A nearly regular mesh of triangles approximates each cerebral hemisphere with a finite edge length of 3.9761 mm on average (Spiegler and Jirsa, 2013, their Fig. 2 and Table 2). The used Gaussian kernel for the homogeneous SC is sampled in the model through the cortical mesh. Because of the finite edge lengths in the mesh, the spatial range of the homogeneous SC should not fall below 6.627 mm for the −3 dB cutoff of spatial frequencies with respect to their magnitude (Spiegler and Jirsa, 2013, their Table 7). The lower bound of the spatial range of σ = 10 mm for the homogeneous Gaussian connectivity kernel causes a loss of at least 20% of spatial information (mainly short range), which corresponds to a −7.13274 dB cutoff (Spiegler and Jirsa, 2013, their Fig. 3A).

### Cellular automaton

The transient period after stimulation onset caused by the transmission times among the 190 brain areas (74 cortical and 116 subcortical areas) in the heterogeneous SC is estimated using a cellular automaton. We use the cellular automaton as a tool to determine a time period for the data decomposition. We focus on the time-delayed interaction among the cerebral areas in the cellular automaton, because the transmissions via the homogeneous SC (short range) of the nodes are instantaneous in the network model in contrast to the heterogeneous SC (long range) of areas, which are composed of at least one node. Each of the 190 cells in the cellular automaton describes one of the brain areas given by the homogeneous SC to be either active or inactive. The temporal decomposition of the heterogeneous SC according to the transmission times gives rules for changing the state of cells over time. The cellular automaton is initialized from the overall inactive state. An activation of a cell triggers a cascade of activation in time until no more cells get activated. In this manner, 190 characteristic activation cascades emerged, each by stimulation, that is, activation of a single cell. The time that the cellular automaton enters the steady state across all stimulation estimates the transient period from the time delays in the heterogeneous SC. This estimate of the cellular automaton was then used to set the starting time for decomposing the simulated data of the full model (Eqs. 1, 2).

### Stimulation and decomposition

All network nodes of a brain area are constantly stimulated for a period of the characteristic time of the nodes, *η*^−1^, to evoke damped oscillations with a maximum magnitude of one. The stimulation response of an isolated node is subtracted from the response of stimulated nodes in the network. A principal component analysis (PCA) was performed using the covariance matrix among the 16,500 nodes. The period of 0.5 s of data after 0.5 s of stimulus onset (estimated by the cellular automaton) was decomposed. For further analysis, up to three principal components (i.e., orthogonal) are considered that cover >99% of variance across conditions.

### Subspace similarity, clustering, and responsive networks

The dot product of the normalized eigenvectors from the decomposition of the stimulation response was used to measure the similarity of the dissipation across different stimulation sites for a range of values of the balance of the SC and a spatial range of the homogeneous SC. The eigenspaces are clustered based on the similarity measure using k-means for each SC balance and each range of the homogeneous SC. The number of clusters is estimated via the gap statistic (Tibshirani et al., 2001). For each cluster, the eigenspaces are rotated to the basis of the one with the highest similarity among all in the cluster, using the singular value decomposition and calculating the optimal rotation matrix (Kabsch, 1978). Averaging the aligned basis vectors in a cluster (across eigenspaces) gives the set of eigenvectors for each cluster. Each resulting eigenvector indicates the contribution of each network node (e.g., whether it belongs to a cortical or a subcortical structure) to a dynamically responsive network.

### Statistics on dynamically responsive networks

A Kolmogorov–Smirnov test is performed to determine whether the cortical and the subcortical contributions to a dynamically responsive network are drawn from the same distribution. A Wilcoxon rank sum test is used to determine whether the cortical and the subcortical contributions to a responsive network are equivalent. A significance level of 0.01 is used for both of these tests.

### Comparing dynamically responsive networks and RS networks

Guided by the Brodmann area designation of the Automated Anatomical Labeling Template (Tzourio-Mazoyer et al., 2002), the cartographic description of the RS networks by Damoiseaux et al. (2006) is mapped onto the geometrical model of the cortex, and its parcellation is used here to determine whether networks that are dynamically responsive to stimulation resemble the experimentally known spatial activity patterns at rest. In the study by Damoiseaux et al. (2006), cortical structures are either mentioned or are explicitly emphasized to be part of an RS network, but are not explicitly excluded. For the present purposes, we assumed areas that were not mentioned were also not part of an RS network. Finally, in the time since their 2006 publication, there have been a number of updates to the functional designation of the different RS networks. We have kept the original designations save for the “unspecified” RS network, which seems to best correspond the dorsal attention network (Cole et al., 2010).

The resultant map onto our geometrical model describes the probability of an area to contribute to an RS network by the following three levels: no, medium, or high contribution for unmentioned, mentioned, or explicitly emphasized (Damoiseaux et al., 2006). The Bhattacharyya (1946) coefficient is then used to estimate the amount of overlap (i.e., the square root of the inner product) between an RS and a dynamically responsive network, the elements of which are essentially indicated by an eigenvector. The square of each eigenvector element is taken and summed up within each area. The coarse-grained eigenvectors and each sum of a combination thereof (four in total) are normalized to unit length. RS networks and responsive networks are compared using the Bhattacharyya coefficient *BC* for an RS network and each normalized coarse-grained eigenvector or combination thereof. The significance of each comparison, *p* = (*n* + 1) /(*N* + 1) is estimated by *N*-times permuting the entries of an RS network (without replacement), calculating the coefficient, BC^ (the permuted Bhattacharyya coefficient), and then counting the values greater than the original, *n* : BC^i > *BC*, with *N* = 2 × 10^6^. The *p* values are corrected due to 24 independent multiple comparisons (eight RS networks with three eigenvectors per stimulation site), using the Bonferroni–Holm correction. A *BC* with *p* values <0.05 is considered to be significant. The mean across the maximum significant overlap for the RS networks with a responsive network (i.e., a single eigenvector or a combination thereof) gives the optimal parameters for (1) the used eigenvector-coarsening metric (i.e., absolute or squared value), (2) the balance of the homogeneous SC and the heterogeneous SC, and (3) the spatial range of the homogeneous SC. The optimum parameter set is separately determined for all the dynamically responsive networks to cortical, subcortical, and both cortical and subcortical stimulations.

### Comparing dynamically responsive networks and connectivity structure

**Figure 4.**
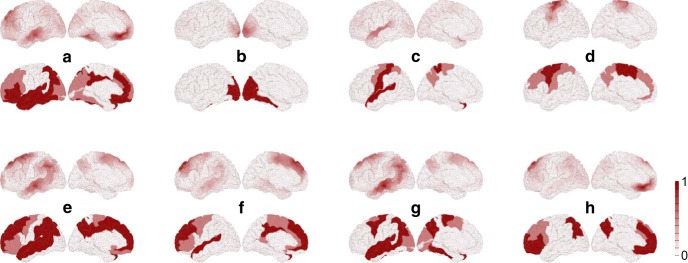
Comparison between dynamically responsive networks to stimulation (top rows) and the experimentally observed RS networks (bottom rows) for the lateral and medial surface of the brain. ***a****–****h***, Default mode, visual, auditory-phonological, somatomotor, memory, ventral-stream, dorsal attention, and working memory. We used 20% to 80% for the ratio of heterogeneous/homogeneous SC and a range of 10 mm for the homogeneous SC. The white to red scale gives the relative contribution of areas to the responsive networks (top rows) and the RS networks (bottom rows). The stimulation sites are given in Table 2 and Figure 7. Note that the bottom rows are activity masks for the 74 cortical areas constituting the RS networks, where activity is not localized within areas and uniformly color coded (see Materials and Methods). The top rows show the vector field Ψ (*x*, *t*) on the mesh of 16,384 cortical nodes and thus localized activity.

A dynamically responsive network is measured by means of contributing network nodes after stimulation (i.e., an eigenvector). The spatial structure (in each eigenvector) is specific to each of the dynamically responsive networks that best explain an experimentally observed RS network (Fig. 4). The eigenvectors corresponding to these eight dynamically responsive networks are compared to the heterogeneous SC. Because this SC describes the wiring between brain areas, the role of each brain area within the network is characterized using measures from graph theory, namely, the following: in-, out-, total-degree; in-, out-, total-strength; and clustering coefficient (Rubinov and Sporns, 2010). Incoming, outgoing, or all connected ties to an area are measured in terms of (1) their numbers and (2) their weights. By counting the connections, we obtain the in-, the out-, and the total-degree. By calculating the sum of connection weights, we obtain the in-, the out-, and the total-strength. The clustering coefficient measures the degree to which areas in a graph tend to group together. Each of the seven measures of the brain areas in the heterogeneous SC is then compared with the elements of each dynamically responsive network (i.e., the eigenvector), using the *BC*. To test statistical significance, the same permutation test is used for the comparison of the dynamically responsive networks with the RS networks.

## Results

Following stimulation of a cortical area at rest [i.e., Fig. 2*a*, subcritical regime (e.g., parameter configuration *γ*_1_)], the induced activity initially spreads radially from the stimulation site across area boundaries (Fig. 3*b*, period 0 < *t* < 640 ms), due to short-range and homogeneous SC. Then, propagation occurs across long distances through the brain network via long-range and heterogeneous SC (Fig. 3*b*, period *t* ≥ 640 ms), that is, white matter tracts. In contrast to the radial propagation behavior, which is similar for all cerebral stimulations, nontrivial propagation behavior occurred that is specific to the location of stimulation. The latter observation can alone be attributed to the weights and time delays of connections described by the heterogeneous SC (Fig. 1*e*), which forms the propagation in synergy with the homogeneous SC. Thus, stimulation of adjacent brain areas may cause totally different propagation patterns, as demonstrated by simulating three different cerebral areas in the whole-brain model in Figure 3*b*. Conversely, stimulation at two remote sites may lead to a similar spatiotemporal pattern after an initial transient (Fig. 3*b*, time frame 890 ms). We conclude that the dissipation of the activity induced by the stimulation of different sites can resolve in the same pattern through particular processes formed by the SC. The radial propagation behavior allows the separation of similar network patterns by their formation starting from different sites.

### Dynamically responsive networks

**Figure 5.**
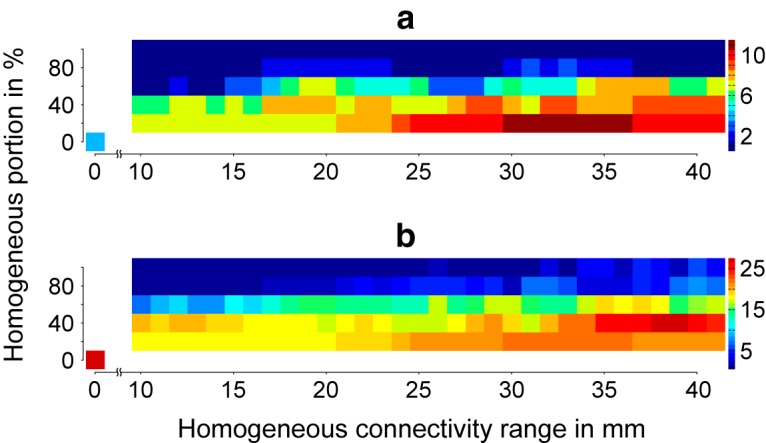
Repertoire of dynamically responsive networks. ***a***, The number of networks responsive to cerebral stimulation depends on the spatial range of the homogeneous SC and the ratio of homogeneous SC to heterogeneous SC. ***b***, Similar to ***a*** for the number of effective cerebral stimulation sites leading to different networks.

From the decomposition of the response activity to a particular stimulation, we obtain three spatially different patterns capturing >99% of the energy dissipation and describing three dynamically responsive networks per stimulation. Regarding our parametric study, we find a maximum of 11 different responsive networks across all cerebral stimulation sites for a ratio of 80% heterogeneous SC to 20% homogeneous SC and a spatial range for homogeneous SC between 30 and 35 mm (Fig. 5*a*). Note that the patterns of these responsive networks are not simply spread activity around the site of stimulation (i.e., radial propagation). With a network of pure heterogeneous SC, only four responsive networks to cortical stimulation can be identified, while the number of responsive networks decreases as the proportion of homogeneous SC increases (Fig. 5*a*). This result supports the synergy of homogeneous and heterogeneous SC in the formation of the network patterns versus a predominant formation via heterogeneous SC. We find a maximum of 27 effective stimulation areas in two occurrences: a 60% to 40% heterogeneous/homogeneous SC ratio and a spatial range of 38 mm for the homogeneous SC; and a 100% heterogeneous SC (Fig. 5*b*). Note that these occur as a result of the stimulation of specific cerebral areas, which lead to the different responsive networks counted in Figure 5*a*. We conclude that, although a pure heterogeneous SC can carry several dynamically responsive networks, considering homogeneous SC dramatically increases the repertoire of networks responsive to stimulation. However, there is an optimal value, as too much homogeneous SC is detrimental to the richness of the repertoire.

### Dynamically responsive networks and RS networks

The decomposition of the response to stimulation of a particular brain area in the whole-brain model resulted in a description of three responsive networks per stimulation. We thus assessed (1) whether these functional networks correlate with the experimentally observed RS networks (Damoiseaux et al., 2006), and, if so, (2) whether the set of RS network patterns do mainly stem from the stimulation of specific cortical, subcortical, or both brain structures. Interestingly, the optimal ratio of heterogeneous/homogeneous SC is found to be 20% to 80% consistently for all stimulation conditions. The spatial range for the homogeneous SC is found to be 10 mm for the two groups of networks responsive to cortical stimulation, and to both stimulation cortical and subcortical. A spatial range of 17 mm was found to be optimal for the group of networks responsive to subcortical stimulations. The locations of the stimulation that are most likely to support energy dissipation into one of the RS network patterns are listed in Table 2 [with its corresponding correlation (Bhattacharyya) coefficient] for each stimulation condition and for the optimal parameterization. Note that a location may appear repeatedly for the same stimulation condition, because the activity after stimulation is decomposed into three orthogonal eigenvectors describing three dynamically responsive networks, where each of which may relate to a different RS network [e.g., area nucleus anterior dorsalis thalami (AD) in thalamus].

**Table 2:** The stimulation sites corresponding to the dynamically responsive network that best match a particular RS network

Resting-state network	Stimulation condition		
	*Cortex* (excluding subcortex)	*Subcortex* (excluding cortex)	*Cortex and subcortex*
Default mode	PFCm (0.8337)	AD (0.8420)	AD (0.8506)
Visual	CCs (0.6455)	GL (0.6953)	GL (0.7510)
Auditory-phonological	TCs (0.7147)	GMPC (0.6630)	TCs (0.7147)
Somato-motor	M1 (0.8153)	MDDC (0.8199)	M1 (0.8153)
Memory	V2 (0.8646)	MDDC (0.8454)	V2 (0.8646)
Ventral stream	CCa (0.7845)	ML, AN, SG (0.8122)	CCa (0.7845)
Dorsal attention	M1 (0.7039)	R, VA, X (0.7097)	AD (0.7631)
Working memory	CCs (0.8006)	PAC, Cdc (0.8204)	GL (0.8069)

All responsive networks of a parameter configuration were compared to the eight experimentally known RS networks. A permutation test was performed to test the significance of each comparison. The multiple comparisons were corrected using the Bonferroni–Holm correction. For the comparison, the dynamically responsive networks were differentiated into: cortically, subcortically responsive networks, and the union of all responsive networks irrespective of the stimulation site. For each of these three groups separately, the parameterization was found to show the best accordance of stimulation responsive networks with the entire set of RS networks. The optimal parameterization is the ratio of 20% to 80% for the heterogeneous/homogeneous SC and a range of 10 mm for the homogeneous SC for all groups, except the range is with 17 mm different for the group of responsive networks to subcortical stimulation. Note the presence of cortical and subcortical sites in the last column, which has higher matching values on average over the eight RS networks compared with the other groups. The value in parenthesis is the matching coefficient (it varies between 0 and 1). Abbreviations are listed in Table 1.

Irrespective of the restrictions to the stimulation (i.e., cortical stimulation, subcortical stimulation, and both), the default mode and the memory network always show the highest correspondence with the dynamically responsive networks, whereas the visual and the auditory networks show the lowest correspondence (Table 2). Moreover, we averaged the best significant coefficients (Table 2) over the eight RS networks to assess whether the set of RS network patterns is driven by (1) cortical areas; (2) subcortical areas; or (3) both cortical and subcortical areas, where a particular pattern is either driven cortically or subcortically. Considering the overall correspondence, the set of RS network patterns is equally well explained by stimulating subcortical sites (*<BC>* = 0.77 on average) than cortical sites (*<BC>* = 0.77), but by stimulating a mixture of both cortical and subcortical sites the mean Bhattacharyya coefficient is higher (*<BC>* = 0.79). The dynamically responsive networks matching best with the RS networks are shown in Figure 4.

**Figure 6.**
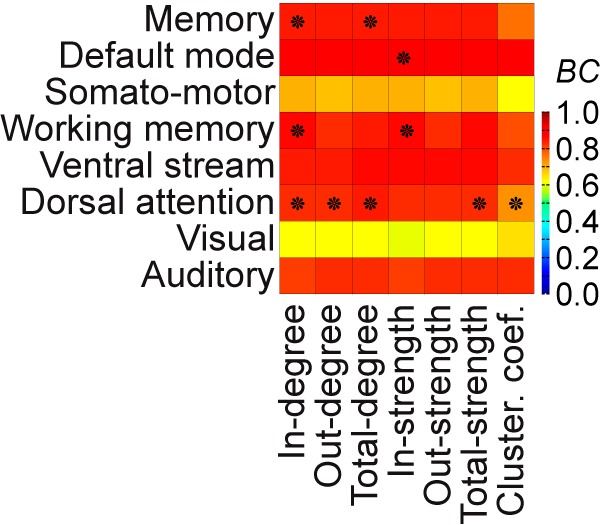
Influence of the structure on the RS-like networks. The pattern of each stimulation-responsive network (from Fig. 5) that best explains an experimentally observed RS network (rows) is correlated with the underlying heterogeneous SC using seven graph-theoretic measures (columns). Incoming, outgoing, or all connected ties to an area can be measured in terms of number (i.e., in-, out-, total-degree) or in terms of strength (i.e., in-, out-, total-strength). The clustering coefficient measures the degree to which areas in a graph tend to cluster together. *BC* indicates a matching with warmer colors, where comparisons marked with a star are statistically significant. Note that correlations may be high but not significant using a permutation test. The in-degree of the heterogeneous SC can be related to the two memory networks and the attention network. The activation of the other RS networks emerges in a way that is not predicted by the network metrics.

To assess whether a dynamically responsive network reflects the underlying structure, we correlated the activity pattern indicating a dynamically responsive network with graph measures of brain areas in the network of heterogeneous SC (Fig. 6). Across the different measures, the in-degree of the SC can be related to the two memory networks and the attention network. For these RS networks, this means that the in-degree of brain areas given by the SC indicated the criticality of areas in the operating large-scale brain network model (Kunze et al., 2016), where criticality is the distance of the operating point of a network node to its inherent bifurcation.

### Stimulation lookup table

**Figure 7.**
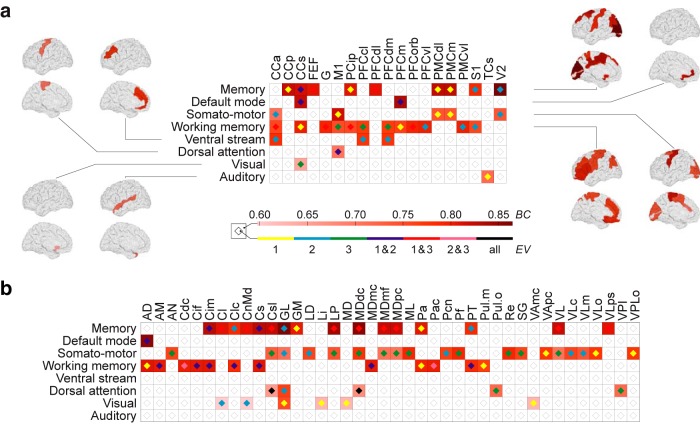
RS-like networks triggered by stimulation. ***a***, ***b***, Cortical stimulations in ***a***, and subcortical stimulations in ***b*** lead to dynamically responsive networks correlating significantly with RS networks for a ratio of 20% to 80% of the heterogeneous/homogeneous SC and a range of 10 mm of the homogeneous SC. *BC* = [0, 1] indicates a matching with higher values. The eigenvectors, *EV* (1–3 in descending order of eigenvalues and captured variance), indicate the responsive networks to an effective stimulation matching with RS networks. Abbreviations are listed in Table 1. Note that the sites triggering a particular pattern can be scattered over the cerebral hemispheres (e.g., for the two memory networks and the somatomotor network).

The dynamically responsive networks can be characterized in terms of stimulation sites, including the responsive networks that resemble RS network patterns. Assuming a direct link between the spatial activity patterns formed at rest (i.e., RS networks) and the task-related functional networks (e.g., related to an external input such as a light flash), RS networks hence can be characterized by the stimulation of particular structures that can be part of (1) a network in which information is processed, (2) an ascending path of sensory input, and (3) structures modulating the processing of a certain input (Fig. 2*d*). All stimulation sites for cortical and subcortical areas in which their responsive networks significantly match with an RS network pattern in our model are summarized in Figure 7. For example, the pattern for the visual RS network is highly responsive to stimulation of the nucleus geniculatus lateralis thalami (GL), which is part of the visual pathway. Considering cortical stimulation, the same pattern is simply activated by stimulation of the Gyrus cinguli subgenualis (CCs), which has been associated with emotion processing and the pathogenesis of mood disorders (Mayberg et al., 2005). Hence, the stimulation of this cortical area modulates information processing in the visual system rather than directly affecting the processing, such as that indicated in Figure 7*a* in the case of the default mode and the two memory networks. According to our study of a large-scale whole-brain network model, thalamic stimulations result in activity most prominently in the following four RS network patterns: default mode, motor, working memory, and the attention network. Cortical stimulations, in particular superior temporal, primary motor, secondary visual, and anterior cingulate cortex result in activity most prominently in the remaining RS network patterns, namely auditory-phonological, somatomotor, memory, and ventral stream network. Note that the dynamically responsive network to cortical areas, especially memory, working memory, and somatomotor, are scattered over the cerebral hemispheres (Fig. 7*a*). In addition, Figure 7 indicates which of the three responsive networks matches with an RS network. Considering that the spatial patterns, which describe the dynamically responsive networks, capture the dissipation of induced network activity after a specific stimulation (in descending order with the variance), we found the following RS network patterns to be dominant (in terms of variance), thus captured in the first dynamically responsive network: the visual, the auditory, the motor, and the working memory networks. The same is true, to a lesser extent, for the memory, the ventral stream, and the attention network. These RS networks were represented in the specific second dynamically responsive network to stimulation, thus the weaker (in terms of the variance) of the particular responses. Interestingly, we found the default mode network to be particularly flexible and spanned by both the first responsive network and the second responsive network to specific stimulation.

## Discussion

This modeling study shows how to generate and predict both spontaneous and task-related network dynamics. Moreover, it provides an entry point for (1) understanding brain disorders at a mechanistic level; and (2) planning more effective therapeutic interventions (i.e., computational neuropsychiatry; Deco and Kringelbach, 2014), for example, through new targets for brain stimulation. Using a whole-brain model (Fig. 1), which is the freely available default large-scale brain network structure of The Virtual Brain [TVB version 1.4.1 (www.TheVirtualBrain.org)], we systematically activated all possible cortical and subcortical areas with brief stimulation to investigate the brain response as a function of long-range SC, that is, white matter fibers, and short-range SC, that is, intracortical connections. We investigated the SC because information processing in the brain strongly depends upon both short-range (intracortical) and long-range (intercortical) connections (Deco et al., 2015), and because previous whole-brain modeling studies mostly focused on long-range SC (Honey et al., 2007; Ghosh et al., 2008; Deco et al., 2009, 2011; Deco and Jirsa, 2012; Hansen et al., 2015). We parametrically varied the ratio of long-range SC to short-range SC and the spatial range of short-range SC (Spiegler and Jirsa, 2013). We obtained the responsive networks by analyzing the energy dissipation of the stimulus-induced activity in the full extent of the structural network (Fig. 3). The focal activations in the large-scale brain model may resemble such invasive stimulation techniques as deep brain stimulation (DBS) (e.g., single DBS pulse; McIntyre et al., 2004; Montgomery and Gale, 2008), and such noninvasive techniques as TMS (e.g., single-pulse and patterned TMS; Dayan et al., 2013). We then contrasted the dynamically responsive networks to functional networks; more precisely, to the eight experimentally known RS networks (Damoiseaux et al., 2006). We found that for a particular configuration of short-range and long-range SC, the network responds to specific focal stimulation with activity patterns that closely resemble RS networks (Figs. 4, 7; Table 2). Moreover, we found short-range connectivity essential for describing RS networks.

Mohajerani et al. (2013) demonstrated in lightly anesthetized or awake adult mice that a palette of sensory-evoked and hemisphere-wide activity motifs is represented in spontaneous activity. Correlation analysis between functional circuits and intracortical axonal projections indicated a common framework corresponding to long-range monosynaptic connections between cortical areas. Mohajerani et al. (2013) also report that most of the robust activation patterns and their evolution appeared long after stimulation, reflecting that the initial dynamics are determined by the local interactions and the stimulation site, but the later developments are shaped by the interplay of connectome and dynamics. These results converge with our findings and suggest that a polysynaptic connectome shapes the spatiotemporal evolution of spontaneous cortical activity.

In the following, we will discuss the model and the simulation results in more detail.

Large-scale brain network modeling succeeded under autonomous situations (e.g., driving the model with noise) to describe the functional connectivity dynamics of ongoing spontaneous brain activity (Honey et al., 2007; Ghosh et al., 2008; Deco et al., 2009, 2011; Deco and Jirsa, 2012; Hansen et al., 2015). The previous large-scale network model studies mostly considered long-range SC, that is, white matter tracts. Here, we went beyond this and incorporated short-range SC to understand how activity propagates and dissipates in the brain (Jirsa and Kelso, 2000; Jirsa, 2004; Qubbaj and Jirsa, 2007, 2009). Time delays arose from the heterogeneous long-range SC. Because of finite transmission speeds, time delays in the short-range homogeneous SC may add dynamics to the network repertoire. The incorporation of these time delays is, however, challenged by the vast number of connections (e.g., 40,597,165 connections in our model, for a characteristic range of 10 mm for the short-range SC), with that the computational expenses, and is considered for future work.

### Brain dynamics and criticality

Brain activity and its functional connectivity (FC) are fluctuating at rest (Allen et al., 2014). FC is thus dynamic and unfolds the SC partially at a given time. To investigate the dynamically responsive networks to focal stimulation, we hypothesized that networks operate at the brink of criticality. So far, predictions from large-scale brain network models related to near criticality have been tested only in autonomous situations of ongoing spontaneous brain activity (Honey et al., 2007; Ghosh et al., 2008; Deco et al., 2009, 2011; Deco and Jirsa, 2012; Hansen et al., 2015). In nonautonomous situations, such as following stimulations of individual brain areas, near criticality, which is linked mathematically to the local center manifold theorem (Haken, 1978), predicts that the poststimulus dynamics evolve with characteristic features in space and time, as follows: (1) the existence of a low-dimensional set of dynamically responsive networks; and (2) their slow decay times after stimulation relative to other networks. This approach provides not only a link among brain stimulation, functionally relevant networks, and RS networks (as suggested by Fox et al., 2014), but also gives a better understanding of the relation between external inputs (e.g., sensory) and internal brain states.

We parameterized the model to operate close to criticality (Fig. 2). The criticality in our brain network model essentially depends on (1) the distance of the operating point of the node to the bifurcation, (2) the effects of the SC on the operating point of the nodes, (3) the ensemble of signal transmission delays, and (4) the stimulation. Though the SC gives a brain specific topology, the model does not show fluctuations at rest, that is, in the absence of external inputs (i.e., no perturbations such as noise or stimulation). Instead, the network is simply silent without a drive and expresses its activity by virtue of stimulation (processing of inputs) by means of damped oscillations. At rest, the operating point of each network node is in the same distance to the critical point, that is, the supercritical Andronov–Hopf bifurcation. Consequently, there is no activity in the network. An excitatory stimulation pushes the network model closer to criticality by selectively moving the operating point of particular network nodes closer to the Andronov–Hopf bifurcation (Fig. 2*a*, from γ_1_ to γ_2_). Because the stimulation is performed on brain areas that are interconnected via the heterogeneous SC, the effect of the stimulation of the network nodes is particular to the site of stimulation. In this way, we have demonstrated that the dynamically responsive brain networks result from near criticality and show the most active and long-lasting patterns following stimulation.

Drivers of brain dynamics can be internal (i.e., autonomous situation) or external (i.e., nonautonomous situation). Considering stimulation as a driver for brain dynamics, white noise is a rather unspecific stimulation with respect to time and space as in the autonomous situations (Ghosh et al., 2008; Deco et al., 2009, 2011; Deco and Jirsa, 2012; Hansen et al., 2015). One may, however, consider a specific external stimulation (e.g., of a given brain area at a given time) as a particular realization of a random process at a given time. In this context, it is worth mentioning that the characteristics of a random process depend on the level of description regarding the SC. For example, in our cortex model we consider short-range homogeneous SC between adjacent network nodes and long-range heterogeneous SC between brain areas, which comprise several nodes (Fig. 1). A spatiotemporally uncorrelated noise added to the state variables on the level of network nodes will inevitably occur correlated on the level of brain areas. The short-range homogeneous SC smoothes the spatial variance, and the differential operator smoothes over time. This indicated that a random process on the level of large-scale brain networks has to be correlated over space and time. Noise is hence more effective in small structures (e.g., thalamic nuclei). To determine stochastic processes for driving a model, the spatiotemporal correlations of brain signals could be used (Spiegler and Jirsa, 2013 and the citations therein).

Dynamically responsive networks are specific to a set of stimulation sites. Activations of a given brain structure by stimulation lead to a brain response that we characterized by a spatial pattern of activity. The set of specific activation patterns composes dynamically responsive networks. Each dynamically responsive network is a fingerprint of the network structure given a specific set of stimulation sites. We extracted the set of dynamically responsive networks by systematically stimulating the brain areas and then comparing the activity patterns. The responsive networks form a set of different spatial patterns of brain activity and are specific to a set of stimulation sites. The meaning of each dynamically responsive network for information processing in the brain can be discussed with regard to the literature and experimental findings, for example, by comparing the response networks with the experimentally known RS networks.

RS networks can be characterized by the stimulation of particular sites. We demonstrated that RS networks could be specifically activated following the stimulation of specific brain areas. Here, the underlying assumptions are as follows:(1) a direct link between the spatial activity patterns formed at rest (i.e., the RS networks and the task-related functional networks); and (2) the emergence of these functional networks from the large-scale brain structure. RS networks correlate with functional networks, which are associated during a task with information processing, such as the perception of a visual stimulus (Damoiseaux et al., 2006). For instance, the FC of the RS networks has been correlated with the SC of white matter tracts (Greicius et al., 2009; van den Heuvel et al., 2009; Hermundstad et al., 2013).

The RS networks formed a subset of dynamically responsive networks. In other words, we found more responsive networks than RS networks. This indicates that functional networks are not restricted to the experimentally known RS networks we considered in this study. These eight RS networks were consistent (and showed the least variation around the mean) across 10 healthy subjects (Damoiseaux et al., 2006). This, however, does not suggest that there are no other, more variable but stable patterns of activity. For instance, the performance of a perceptual task could be related to the individual variability in FC at rest (Baldassarre et al., 2012). The way humans approach and perform the same task can be diverse (Sporns and Edelman, 1993) and involve a variety of functional processing. The task and its complexity may concern functional patterns and networks that vary across and within subjects (e.g., on a trial-by-trial basis). Functional networks are not confined to the experimentally known RS networks. This applies to dynamically responsive networks in the model with regard to RS networks also. One could also argue that brain stimulation (e.g., deep brain stimulation) of a particular brain structure resolves in an activity pattern that is distinct from known (task-related) functional networks and RS networks simply because the stimulation directly affects a targeted brain structure and does not necessarily ascend a sensory pathway (such as a light flash), thus not processed in (and related to) the known task-related functional networks. Consequently, the responsive networks that do not match a known functionally related network pattern may reflect (1) less dominant/frequent networks, (2) functional networks that are not directly related to a task but modulate information processing, or (3) activation patterns that are specific to direct brain stimulation. The role of the stimulation site becomes even more apparent from the detailed analysis of corticocortical SC revealing lateral, ascending and descending projections (Felleman and Van Essen, 1991); thus, a hierarchical organization in which complex interactions, including feedforward, feedback, and parallel processes are supported (Bressler, 2008). A direct link between the RS networks and the task-related functional networks allows the characterization of RS networks by the responsiveness to stimulation of particular structures that are part of (1) networks in which information is processed, (2) ascending paths of sensory inputs, and (3) structures modulating the processing of a certain input (Fig. 2*d*). RS dynamics originate from subspaces, in which the ongoing activity evolves and alters, giving rise to nonstationarity, as observed in empirical and computational studies (Allen et al., 2014; Hansen et al., 2015). Our study predicts that these subspaces can be selectively targeted to bias the brain dynamics toward the activation of specific functional (task-related) and RS networks through stimulation of specific brain areas, for instance, by sensory stimulation (e.g., auditory, visual) and brain stimulation techniques (e.g., transcranial magnetic stimulation). The stimulation sites are predicted to be network specific and spatially clustered but distributed (Fig. 7). Stimulating different brain areas could lead to similar activation patterns during rest conditions.

### Dynamically responsive networks and the underlying SC

The SC mostly predicts the activity of brain areas directly after stimulation. However, as time evolves, both implemented types of SC, short-range (homogeneous) SC and large-scale (heterogeneous) SC, play a crucial role in the spatiotemporal progress. The connectome and its large-scale heterogeneous SC can explain some, but not all, stimulation responsive networks that fit the experimentally observed RS networks best (Fig. 6). Considering the applied network metrics, it is interesting to note that the default mode and the memory networks strongly related to the local embedding of nodes in the topology of the SC, which suggests that they play a special role in information processing. The activation of the other RS networks depends to a lesser degree on the local topologies in the SC and may thus constitute an emergent dynamic process. Emergent properties can be understood by the transmission and synchronization behavior of the oscillatory activities throughout the propagation in the network, which decelerates or accelerates the dissipation process in parts of the network. It has been shown that nodes linked to a network traverse a node-inherent particular bifurcation (e.g., supercritical Andronov–Hopf bifurcation) with scaling the connectivity in the order of the in-strength of the nodes in the underlying structural connectivity (Kunze et al., 2016). This is simply applicable to the two memories and the attention RS networks (Fig. 6) in terms of the criticality of nodes, that is, the distance of the operating point of nodes to its bifurcation point. The comparison with the SC (Fig. 6) indicates that the dissipation processes are sequences of multiple iterations of the SC, and thus over several cycles of damped oscillations, where delays and synchronization naturally play a major role.

Our simulations show that the repertoire of dynamically responsive networks is the richest for the mixed case in which large-scale heterogeneous and short-range homogeneous SCs are simultaneously present (Fig. 5), which is in keeping with known statistics of synapses within a population, namely 50% of intracortical and 50% of corticocortical fibers (Braitenberg and Schüz, 1998). The maximum number of different dynamically responsive networks to cerebral stimulation appeared for a ratio of heterogeneous/homogeneous SC of 60% to 40%, where the number of effective cerebral stimulations is maximum for a ratio of 80% to 20%. Interestingly, considering all stimulation sites, the dynamically responsive networks resembled the RS networks best for a different ratio of heterogeneous SC to homogeneous SC, namely of 20% to 80% and a spatial range of the short-range homogeneous SC of 10 mm. The number of different responsive networks to cerebral stimulation is small (Fig. 5*a*), which may indicate the leading role of thalamic structures at rest and the constrained repertoire of dynamics at rest. The parameter values for the SC characterized the whole-brain network, and thus were similar for all network nodes and areas, but it is likely that they are brain area specific (Felleman and Van Essen, 1991). However, we did not perform an area-specific optimization, as the number of possibilities makes it computationally intractable at the current time. Furthermore, the effects of stimulation on the brain depend not only on the location of the stimulation, its intensity, and its duration, but also on the dynamic state of the brain (Dayan et al., 2013). Large-scale brain network models could be used to describe state dependencies of brain responses (e.g., event-related potentials), including experimental paradigms (e.g., oddball). Not only could the synaptic connections be better adapted to predict the empirical data, but there are also possibilities for improving the characteristics of the local dynamics in each brain area. At the moment, the regional local dynamics are considered homogeneous as a matter of simplification, but could be extended to deal with different heterogeneous local dynamical nodes, for instance, derived from the temporal information in functional data (Deco and Kringelbach, 2014). Furthermore, the spatial range of the homogeneous SC was found at the lower boundary of the studied range. Because the lower boundary depends on the geometrical model of the cortex, a systematic investigation of the effects of cortex resolution, and with that the approximated homogeneous kernel on large-scale brain dynamics, as suggested by Spiegler and Jirsa (2013), is desirable and crucial for the incorporation of local and homogeneous SC in a large-scale brain network model.

Our model can also be used to study the propagation of hippocampal sharp-wave ripples (Logothetis et al., 2012) by describing (1) faster and slower rhythms, (2) the hippocampal formation (CA1, CA3, dentate gyrus) in more detail (including its specific SC), and (3) specific states (e.g., slow-wave sleep and anesthesia). This could provide an entry point for investigating memory consolidation, changes of brain states, and its functional networks. However, the stimulation of the hippocampal cortex (HC) activated no RS networks (Fig. 7). This study should also serve as a good starting point to investigate repetitive stimulation (e.g., with respect to deep brain stimulation; Murrow, 2014) and the spatiotemporal dynamics of brain resonance phenomena (Spiegler et al., 2011).

In conclusion, we demonstrated that that short-range connectivity proves beneficial in whole-brain network models for describing brain activity. Moreover, we demonstrated that a large-scale brain network dissipate their energy spatiotemporally upon stimulation in a characteristic low-dimensional manner, which is consistent with the idea that the brain operates close to criticality. The stimulation-responsive networks are compatible with the empirically known RS networks and are set apart by the slow time scale as predicted by theorems of near criticality. Stimulation sites can be assembled in topological groups that approximate empirical RS networks. A stimulation of brain areas in these groups predicts an evolution of the RS dynamics toward lower-dimensional subspaces, in which the subsequent dynamics evolve and can be characterized by conventional FC approaches. Our results suggest a means to bias RS dynamics via spatially coordinated stimulation toward target subspaces. Given that the FC of the RS differentiates groups with different pathologies and across ages, our results are of interest for approaches of such brain stimulation techniques as transcranial electrical stimulation, transcranial magnetic stimulation, and deep brain stimulation directed toward therapy and cognitive enhancement.

## Synthesis

Reviewing Editor: Gustavo Deco, Universitat Pompeu Fabra<br />Decisions are customarily a result of the Reviewing Editor and the peer reviewers coming together and discussing their recommendations until a consensus is reached. When revisions are invited, a fact-based synthesis statement explaining their decision and outlining what is needed to prepare a revision will be listed below. The following reviewer(s) agreed to reveal their identity: Raphael Kaplan, Rosalyn Moran. For some manuscripts transferred from JNeurosci, a decision is made to accept the manuscript without peer review and a brief statement to this effect will follow.

Both Reviewers were positive and are asking in my opinion for minor changes.

Reviewer 1:

This is a sophisticated analysis of whole brain networks and RSNs using the virtual brain platform. In order to provide a significance advance (understanding why and/or if RSNs emerge from constrained topologies) the authors should re-organize their analysis and conclusions to clarify the aims of the model analysis it is at times circular.

In their manuscript "Selective activation of resting state networks following focal stimulation in a connectome-based network model of the human brain", the authors test whether nodal stimulation in a given brain region results in the recruitment of known resting state networks.

The study employs the virtual brain platform (TVB) which enables a whole brain simulation based on structurally coupled mean field models of neuronal cell assemblies.

The manuscript is motivated in part by the emerging/growing field of neurostimulation and the need to be able to predict stimulation effects downstream of the target region. The approach using TVB is timely and to be commended.

There are some very interesting findings here including the overlap of dissipation networks for a given stimulation site. However it is often difficult to tell whether findings are imposed by certain simulation constraints or are emergent features/results i.e. it is at times hard to tell what is put into the model and what emerges from the model.

Major comments:

The precise aims of the study are unclear and seem at times, circular. The authors report emergent RSns from different parameterizations of local and global structural connectivity contributions but the reader is not clearly shown whether the RSn are the predominant characteristic or a subset of the 16000+ simulations. Furthermore some free parameters (proportion of long range vs short range connections and expanse of local connections) are optimized in order to produce a maximal number of RSNs.

Thus it seems that the goal of the paper is to determine the optimal connectivity matrix to produce RSNs not to test whether RSNs are the main emergent feature of the simulated dynamics. Thus the authors might consider framing the abstract and title as a search for not proof of RSNs.

Or better still would be to show whether RSNs are a predominant feature across all reasonable parameterizations i.e. those already tested - and then explore the optimum parameter landscape for this feature.

Another alternative is whether this study is designed to test what particular cortical node might result in dissipation networks in a particular RSNs. This is a third distinct aim (presented in fig 7) and should be clearly signposted in the introduction, methods and results.

Thus the following statements in the abstract and introduction are confusing : "If the connectome constrains how brain areas are functionally connected, the stimulation of specific brain areas should produce a characteristic pattern of activity ultimately resolving into RS-networks. To systematically test this hypothesis we use...,". This is not explicitly addressed in the paper.

And in the introduction "the connectome imposes strong constraints upon where and how information will be transmitted and distributed but direct evidence is still lacking." The paper does not provide evidence for or against it described the conditions under which it emerges (and without context of what else is unfolding given a stimulation site).

To be concrete - 190 stimulation sites are tested. However RNS emerge from 18 (figure 7). What happened when the other 172 areas were stimulated?

Methods Description. Some of the methods section was difficult to follow. Would it be possible to refer to previous standard TVB settings and focus on the methods unique to this analysis. For example the full description of the field model.

Results section: Figure 5. Why does the figure not show all 0 % homogenous contribution - just one blue square - what does the blue square indicate? What is the difference between a and b? Neither the text not the caption mention anything other than % contribution from hetero/homogenous SC vs. spatial range.

Line 226 - 276 is difficult to interpret. What does high, medium and low mean? What is the definition of a BC? And why are 24 mutliple comparisons used when 190 stimulation sites are tested? Are 24 tests performed or are 190 x 8 tests performed? If not the latter why not the latter - since you are testing for the emergence of RSNs.

Does the BC describe the contribution of 1 of the 3 eigenvectors? In other words if 2 eigenvectors from the network explained 95% of the variance and the 3rd eigenvectors the remaining 4% then is a positive test of RSN overlap if it overlaps only with the 3rd eigenvector?

Results section :" From the decomposition of the response activity we obtain a maximum of three dissipation networks per stimulation site capturing 99 % of the dissipation". Is this true for every stimulation site or is this the average across all tested sites? How is 'a network' defined ?

Some arguments are a little confusing: The simulation assumes that the 'baseline' state itself is at the edge of criticality. In the introduction though the authors argue that this edge of dynamic stability regime might result in RSNs once stimulated. However 'baseline' states are presumably represented by RSNs themselves. How do the authors square the fact that "the proximity to criticality predicts that the brain's response to stimulation will primarily arise from structures and networks that are closest to instability" with the definition of RSNS as those emerging from no stimulation.

Minor comments:

Some statements are difficult to understand:

Methods: "The region on each sphere where the corpus callosum intersects is simply taken out by placing all nodes in these two regions far enough apart so that the homogeneous SC of these nodes is g ( x - X' ) -> 0. Finally, one region is the intersection by the corpus callosum and the remaining regions are the considered 37 cortical areas composing a cerebral hemisphere.'...Could this be reworded?

'Since the transmissions are assumed to be instantaneous in each cerebral hemisphere among the 8,192 nodes through the homogeneous SC, the cerebral areas and its transmission times are considered to cause transients rather than each single node in cerebral areas and its short-range interactions'.... Could this be reworded?

------

Reviewer2

The authors use a connectome-based large-scale model of the human brain with realistic structural connectivity in order to see how virtual 'stimulation' of specific brain areas propagates to other brain regions. The authors show that depending on stimulation site and frequency, the resulting propagation resembles well-characterized resting-state networks. I'm by no means an expert on the technicalities of neural mass models, such as the Hopf model, so I hope there are other reviewers with that expertise. I still found the paper interesting and think that it will contribute to the literature, especially given the relative dearth of large-scale modelling work. However, I found that the importance of some of the results were unclear and the discussion was a bit lacking in some parts. I think the manuscript would be more useful to the community if the authors addressed these three main concerns before publication.

-I think it would be useful to compare the model with previous research from the lab of XJ Wang investigating virtual stimulation and propagation in the monkey neocortex(see Chaudhuri et al., 2015 Neuron). With a much simpler model than the one presented in this manuscript, Chaudhuri and colleagues found that the key to observing different time scales in different neocortical regions was the anatomical heterogeneity of connection strengths more than the structural connectivity itself. In order for the reader to assess the validity of their Virtual Brain model, I think it is important to dedicate some figure showing a model comparison of how the results depend on whether the CoCoMac connection strengths are changed (e.g. see Fig. 5 in the Chaudhuri paper or maybe show randomised connection strengths versus homogenous connection strengths vs the real connection strengths). With an analysis and discussion addressing the importance (or lack thereof) of anatomical heterogeneity, the reader can better assess the utility of the model/manuscript.

-The authors address the importance of their model for clinical stimulation, but I think they overlook other work by the lab of Logothetis looking at the propagation of hippocampal sharp-wave ripples (and electrical stimulation of the hippocampus) and their influence on BOLD (Logothetis et al., 2012 Nature; Ramirez-Villegas et al., 2015 PNAS) and RSNs (Kaplan et al., 2016 Current Biology) that closely relates to their results. A discussion of this work in the context of their paragraph about Mohajerani et al., 2013 would more clearly highlight the relevance of the authors' findings and possibly provide more predictions that could be made from their model.

-Lastly, this point might end up being related to my first point, but I found it surprising that subcortical and neocortical stimulation ended up having an equal influence. Given the extensive long-range connections of subcortical areas like the hippocampus and thalamus, I'd expect stimulation there to have a greater influence than most neocortical regions. Discussion of the possible mechanism behind this finding is important.
